# Deep learning in light–matter interactions

**DOI:** 10.1515/nanoph-2022-0197

**Published:** 2022-06-14

**Authors:** Daniel Midtvedt, Vasilii Mylnikov, Alexander Stilgoe, Mikael Käll, Halina Rubinsztein-Dunlop, Giovanni Volpe

**Affiliations:** Department of Physics, University of Gothenburg, Gothenburg, Sweden; Department of Physics, Chalmers University of Technology, Gothenburg, Sweden; School of Mathematics and Physics, University of Queensland, St. Lucia, QLD 4072, Australia,

**Keywords:** deep learning, neural networks, optics, photonics

## Abstract

The deep-learning revolution is providing enticing new opportunities to manipulate and harness light at all scales. By building models of light–matter interactions from large experimental or simulated datasets, deep learning has already improved the design of nanophotonic devices and the acquisition and analysis of experimental data, even in situations where the underlying theory is not sufficiently established or too complex to be of practical use. Beyond these early success stories, deep learning also poses several challenges. Most importantly, deep learning works as a black box, making it difficult to understand and interpret its results and reliability, especially when training on incomplete datasets or dealing with data generated by adversarial approaches. Here, after an overview of how deep learning is currently employed in photonics, we discuss the emerging opportunities and challenges, shining light on how deep learning advances photonics.

## 1 Introduction

The interaction of light with matter at the subwavelength scale constitutes the foundation for many applications, ranging from microscopy and nanosensors to data storage and optical communications [1–3]. To optimize the performance of such applications, the ability to predict and analyze light–matter interactions is crucial. Traditionally, these tasks have been based on algorithmically solving Maxwell’s equations for a given setup geometry, whose parameters need to be determined from experimental measurements. However, this approach is often time-consuming, applicable only to relatively simple geometries, and very sensitive to measurement noise [4].

Recently, there has been a surge of interest in employing machine learning, especially deep learning, to tackle the limitations of traditional approaches [5]. Briefly, a deep-learning model is an artificial neural network that converts vectors of input data into vectors of output data through a series of transformations characterized by a large number of trainable parameters [6]. The choice of the structure of the network, i.e., its *architecture*, is still mostly a matter of taste and experience rather than a result of clearly established principles. The network must be sufficiently complex to encode the problem at hand but not so complex as to resist training. Once the architecture is defined, the network is typically trained by employing a set of input data with corresponding desired outputs. Networks with thousands of trainable parameters can be systematically optimized using algorithms such as *stochastic steepest descent* and *error backpropagation* [7] in a reasonable amount of time using commonly available computing resources.

The design of photonic devices by machine learning started back in the 1990s with the optimization of microwave circuit components [8], starting with recurrent neural networks [9] and then transitioning to feed-forward neural networks [10]. Later, in the early 2000s, neural networks were used to design photonic crystal fibers [11, 12]. In the last decade, there has been a tremendous rise of attention to neural networks for the inverse design of photonic and plasmonic components [8], including photonic crystals [13–17], layered photonic structures [18–22], radiation cloaks [23–25], diffractive optical elements [26, 27], metasurface-based devices working in different bandwidths [28–47], and nanoparticles [48–50]. Apart from designing photonic structures, deep learning has also been successfully deployed to interpret light–matter interactions, e.g., for microscopy [51] and optical data storage [52]. Beyond this, recent works have demonstrated all-optical implementations of deep-learning networks, enabling even faster execution times [53].

Despite this widespread interest, successfully deploying a deep-learning solution is still non-trivial. The strength, and weakness, of deep learning is that the user does not provide the rules connecting the input data to the desired outputs. Instead, the system learns these rules by being fed with ground truth input/output pairs and iteratively adjusting its internal trainable parameters until it reliably provides the intended outputs for the training cases. This enables deep-learning-powered approaches to learn to solve specific problems with the utmost efficiency. However, the lack of user-specified rules also makes it difficult to assess the robustness of the performance of a deep-learning model when presented with data that differ significantly from the training set.

This review provides an overview of the recent success stories and opportunities for applying deep learning in photonics while highlighting the most common challenges and pitfalls encountered when solving a problem using deep learning. In Section 2, we will review some of the most common building blocks and architectures for deep-learning-enabled optics and photonics, as well as the most commonly encountered concepts when working with deep learning. In Section 3, we will review some of the main success stories in applying deep learning to optics and photonics. In Section 4, we will provide an overview of the areas where we believe that photonics and deep learning can work synergistically to offer novel opportunities. In Section 5, we will review the essential challenges and provide simple guidelines for effective deployment of deep-learning-based techniques to study light–matter interactions.

## 2 Current approaches in deep-learning-enhanced optics and photonics

**Figure 1:**
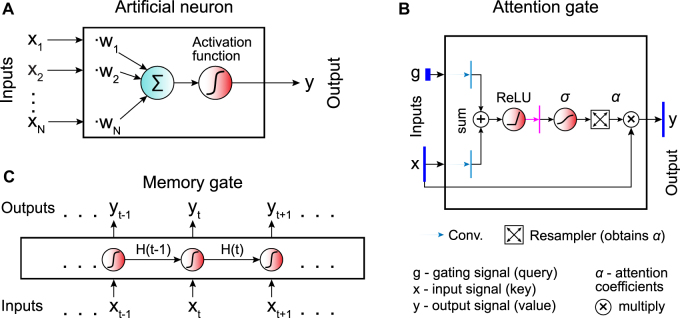
Building blocks of artificial neural networks. **A** The basic unit of a neural network is the *artificial neuron*, which performs the sum of its inputs (*x*_*i*_) weighted by trainable parameters (*w*_*i*_) and applies an activation function to get its output (*y*). **B** Temporal information can be encoded by introducing *memory gates*, which retain memory of their history of inputs. The output *y*_*t*_ at the time step *t* depends on both the current input *x*_*t*_ and the hidden state *H*_*t*−1_ obtained in the previous step. Neural networks containing one or multiple memory gates are called *recurrent neural networks* (RNNs). **C** *Attention gates* provide a way to detect long-range temporal or spatial dependencies within the data by guiding the network towards the most relevant parts of the input data.

The basic building block of a neural network is an *artificial neuron* (Figure 1A). The artificial neuron performs a weighted sum of inputs and returns a (typically) nonlinear transformation (*activation function*) of the resulting sum. The weights are trainable parameters that are tuned during the learning process to optimize the output [54].

A variant of the artificial neuron is the *memory gate* (Figure 1B). In contrast to the standard artificial neuron, which transforms and feeds forward the information it receives without keeping any memory of it, the memory gate adapts its internal state in response to previous data [55], which is useful, especially when analysing time series. Neural networks containing multiple memory gates are collectively known as *recurrent neural networks* (RNNs).

The *attention gates* provide a more general way to encode dependencies in the input data (Figure 1C), which is often superior to alternative approaches such as RNNs for temporal series and convolutional neural networks for images, particularly for long-range dependencies [56]. The gate receives an input signal *x* and a gating signal *g*. These two signals undergo several transformations, which produce the attention coefficients *α* ∈ [0, 1] representing the relevance of the elements (e.g., pixels in an image) of the input signal. Then, the resampler associates a unique attention coefficient to each element of the input signal. Finally, the attention gate outputs an element-wise product of the attention coefficients and the input signal. This guides the network to interesting areas of the input data (the neural network pays attention to specific regions).

**Figure 2:**
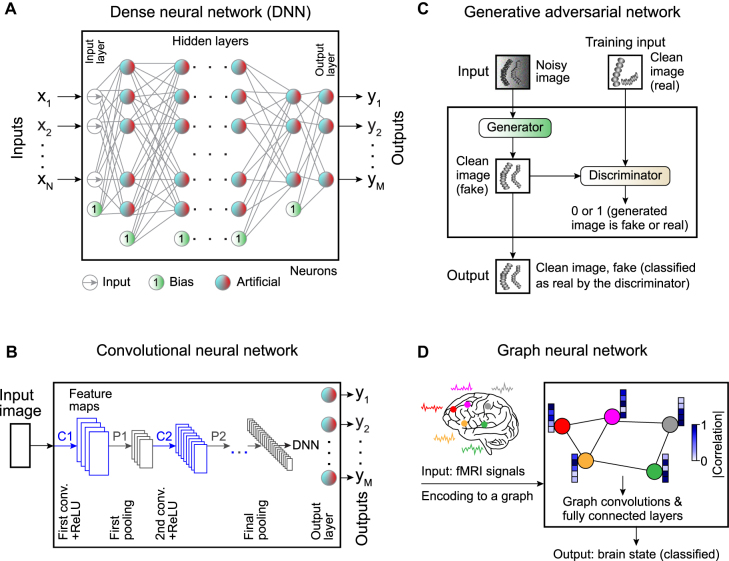
Commonly employed neural networks. **A** Artificial neurons can be combined in a *dense neural network* (DNN), where the input layer is connected to the output layer via a set of hidden layers. All the nodes in each layer are connected to all the nodes in the subsequent layer, hence the name “dense.” There are also bias neurons that add a constant to the weighted sum. **B** Spatial information can be preserved using a convolutional neural network (CNN) with convolutional kernels whose weights are trainable parameters. **C** *Generative adversarial networks* (GANs) provide a framework to generate synthetic data. Synthetic data is generated by passing input data through a neural network (the *generator*), while a second neural network (the *discriminator*) tries to determine whether the data is real or synthetic. The discriminator’s output is used during training to guide the generator to produce more realistic data. **D** *Graph neural networks* (GNNs) provide a powerful method to analyze complex dependencies in the input data using the framework of graph theory.

*Dense neural networks* (DNNs) consist of connected layers of artificial neurons (Figure 2A). All the nodes in each layer are connected to all the nodes in the neighboring layers (*fully connected network*, also referred to in the literature as a *feed-forward neural network* or *multilayer perceptron-based networks* [57]). Usually, DNNs are utilized for data of small dimension. One could also successfully use DNNs in recognition tasks for small images (e.g., 28 × 28 pixel with 10 predicted classes in the MNIST dataset of handwritten digits [57]). However, when it comes to large images containing thousands of pixels, the number of connections between the neuron layers (and the number of learnable parameters) increases drastically, leading to overfitting. Due to the limited number of neurons in the first hidden layer (which is smaller than the number of pixels in a large input image), the amount of data passed from the input through the ANN is limited.

To circumvent these problems, *convolutional neural networks* (CNNs) [58] use partially connected layers made of 2D arrays of neurons (Figure 2B). Each convolutional kernel (*filter*) uses the same weights across different regions of the image, meaning that the weights are shared. The limited number of connections and weight sharing reduces the number of parameters to be trained, thus, lowering the computational load and the chance of overfitting.

A CNN transforms the input image through a large number of filters [57]. Each filter corresponds to a *feature map*. Typically, one filter detects one feature in the picture, and several filters are used in each *convolutional layer* to detect multiple features. The image is typically downsampled several times to access the information at multiple length scales and reduces the computational load, as schematically shown in Figure 2B. Thus, the image gets smaller while passing through the various layers of a CNN [57]. Sometimes, a dense network is appended to the output of the CNN (called a *dense top*) to produce a final output representing the global information related to the input image, e.g., the coordinates of the position of a particle to be tracked [59].

Deep learning has recently been shown to be able also to generate synthetic data of high quality. Generally, such approaches are helpful to generate outputs that are easily interpretable by humans. Deep-learning-enabled generation of synthetic data is typically achieved through *generative adversarial networks* (GANs) [60] (Figure 2C). GANs are deep-learning models with a unique training scheme called *adversarial training* (training competing ANNs), which is one of the most important recent ideas in machine learning [57]. The idea is that the input data (e.g., a noisy microscopy image) is passed through one neural network called the *generator*, which creates the synthetic data (e.g., the corresponding noise-free image). While training the generator, its output is passed through a second network called the *discriminator*, whose task is to determine whether its input is fake or real data. Usually, both the generator and the discriminator comprise several types of ANN architectures, such as dense neural networks, convolutional neural networks, recurrent neural networks, and graph neural networks. At each training step, the generator’s parameters are updated to fool the discriminator. The GAN training consists of many iterations in which the discriminator and generator are both updated in tandem. Within a single training iteration, the GAN update is carried out in two phases [57]. First, the discriminator is trained with the generator’s weights fixed. The fake images (labeled as 0, produced by the generator) and the real images (labeled as 1) constitute the training set in this phase. Second, the generator is trained with the discriminator’s weights fixed. The real images are absent in that training phase. Again, the generator creates fake images. In this case, the generator aims to produce data that the discriminator classifies as true images (all 1s). The error vector is being backpropagated through the discriminator to the generator to update its weights. By iterating these two phases multiple times, the generator learns to fool the discriminator, while the discriminator learns to distinguish true images from fake images. In this way, the output of the generator will look more and more realistic.

As a final example, *graph neural networks* (GNNs) provide a powerful method to analyze complex dependencies in the input data in various physics systems where it is necessary to deal with graph data [61] (Figure 2D). A graph is represented by a set of nodes (the data points) interconnected via edges (corresponding to the dependencies in the input data). Depending on the settings, the task of a GNN can be to classify nodes in the graph, predict edges between nodes from incomplete graphs, or generate entire graphs by training on representative data [62]. For example, GNNs have been actively used in the neural science for classification of the neurological disorders (Figure 2D), where they exhibit greater performance than alternative functional magnetic resonance imaging (fMRI) analysis methods [63, 64], and to track microscopic particles, where they have been able to accurately estimate dynamical properties in various biologically-relevant scenarios [65].

## 3 Success stories

In the past decade, deep learning has found many successful applications within optics and photonics. For most of these applications, the underlying theory connecting the input to the output is unknown or too complex to be of practical use. In such cases, deep learning provides a means to automatize processes that otherwise would require human intervention or large computational resources. In the following sections, we will explore three applications where deep learning has been particularly successful: inverse design of photonic devices, analysis of microscopic and nanoscopic data, and enhancement of microscopy techniques.

### 3.1 Inverse design

The geometry of a nanostructure corresponding to a desired optical response can be optimized by brute-force parameter sweeping. For example, in Refs. [66, 67], cylinder-shaped meta-atoms were optimized by sweeping the cylinder diameter *D* with a fixed height *H* and recording the optical response as a function of the vacuum wavelength *λ* to determine the maximum quality factor (Q factor) at the desired wavelength. This was required to lower the lasing threshold and to realize lasing in the smallest nanoparticle possible [67]. Such a brute-force optimization method takes advantage of the scale invariance of Maxwell’s equations (i.e., multiplying *λ*, *D*, and *H* by the same constant retains the optical response, permitting the authors to employ dimensionless parameters in sweeping the parameter space). A similar optimization method was implemented in Ref. [68], where a pixelated dielectric metasurface was used to record the absorption fingerprint of a protein. The metasurface was made of meta-atoms arrays (pixels). The geometrical parameters of the meta-atom were linearly scaled by a constant factor to get a different reflectance peak position for each pixel. One hundred pixels were stacked to form a metasurface covering the required bandwidth to map the protein absorption. The brute-force sweeping of the parameter space imposes strict constraints and can easily become computationally cumbersome, especially for geometries with many (>3) parameters.

A powerful alternative is provided by inverse design. Inverse design imposes fewer constraints on the investigated geometries [69], which broadens the solution space and results in more efficient devices. However, the larger design space makes numerical simulations more time-consuming. This demands an efficient method for solution space exploration to lower the simulation time. Such methods can be distinguished into traditional inverse design approaches and inverse design with neural networks.

Traditional inverse design methods explore the solution space iteratively, based on a set of rules. The target is to maximize the fitness function (usually a single number), which is evaluated at every step, after which the system’s parameters are adjusted. Stochastic search rules of traditional inverse design methods limit the solution space and the efficiency of the produced devices [69–71]. For example, in Ref. [72], a polarization beam splitter was designed using one of the topology optimization methods known as the *direct-binary search algorithm*. The polarization beam splitter had a square shape (2.4 μm × 2.4 μm), discretized into 20 × 20 square pixels. Thus, each pixel had an area 120 × 120 nm^2^ filled either with silicon or air (1 or 0). An initial binary pattern was randomly generated. The state of a random pixel was switched (perturbed), and the fitness function was computed. The fitness function was defined as the average transmission efficiency for TE- and TM-polarized waves. The perturbed pixel state was kept (retracted) if the fitness function increased (decreased). Then, the state of another pixel was perturbed. One iteration consisted of toggling all the pixels sequentially. The procedure was repeated until the fitness function saturated. Since the outcome is sensitive to the initial guess, several initial patterns were considered to achieve the best design. It took more than five days to obtain one design.

**Figure 3:**
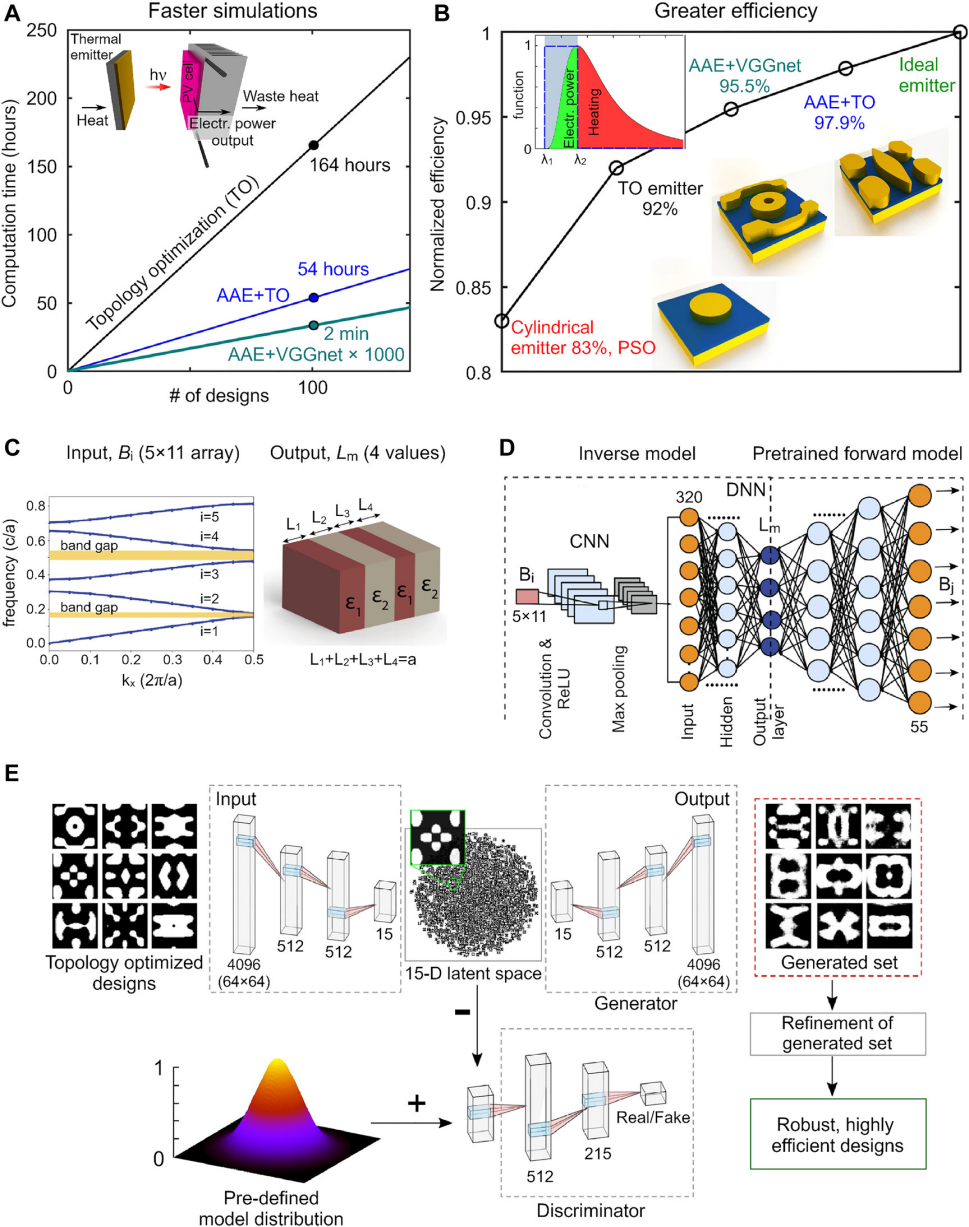
Inverse design of photonic devices by neural networks. **A**, **B** Advantages of neural networks with respect to some conventional simulation methods, namely topology optimization (TO), and particle swarm optimization (PSO). TO is used to generate the training set. TO and adversarial autoencoder (AAE) are compared in the inverse design of the metasurface thermal emitter. TO and CNN VGGnet are employed for structure refinement. **A** Computational time versus number of highly efficient designs. Inset: Schematics of the thermophotovoltaic engine. **B** Efficiencies for the best designs obtained via different simulation methods. Left inset: Normalized spectrum of the black-body radiation; the grey region highlights the photovoltaic cell working band; only the in-band radiation is transformed into electrical power; the blue line depicts the ideal emitter emissivity. Right insets: Finest meta-atom patterns. **C**, **D** Inverse design of a photonic crystal slab. **C** Band structure (left) used for the inverse design of the 1D photonic crystal slab (right). **D** Architecture of the neural networks implemented in the inverse design. **E** Generative model handling the inverse design of the metasurface thermal emitter. Panels A, B, E are adapted with permission from Ref. [73] (Copyright 2020 American Institute of Physics), and panels C, D from Ref. [77] (Copyright 2020 Optical Society of America).

In the case of a machine learning-based inverse design, a neural network is trained using many structures with different geometrical parameters (outputs) and the corresponding computed optical responses (inputs). The trained neural network can then be used to obtain the geometry corresponding to a desired optical response. Inverse design with neural networks has the advantage that it is more time-efficient than traditional methods as it does not require case-by-case simulations [70] (once the neural network is trained). Thanks to this feature, the design of new devices is orders of magnitude faster for neural-network-based approaches than for conventional inverse design methods (Figure 3A) [38, 50, 73, 74]. On the other hand, the computational complexity is moved to the generation of the training set. In fact, the quality and size of the training dataset ultimately determine the quality and accuracy of the neural network output [57, 75, 76].

Another advantage of inverse design with neural networks is that it has fewer restrictions on the considered device geometries [78], as compared to conventional methods (e.g., limitation by pre-defined stochastic search rules [72]). Thus, devices and efficiencies unachievable by traditional inverse design approaches can be obtained (Figure 3A and B) [73, 79, 80].

In recent years, neural networks architectures such as DNNs (Figure 2A), CNNs (Figure 2B), GANs (Figure 2C), and hybrid models have been heavily utilized in photonic inverse design [5, 8, 70, 71, 76, 81–86] (Ref. [8] provides an interesting historical perspective). In photonic inverse design, DNNs can be utilized to predict a finite sequence of values. For example, predicting five values of shell thickness in a multilayer particle, given many points of its scattering cross-section spectrum [50]. Besides, DNNs have been successfully used for inverse design of nanoantennas [50, 86, 87], metasurfaces [25, 88], [89], [90], and grating couplers [91, 92].

CNNs have been heavily used as parts of generative models for the inverse design of photonic structures [70]. CNNs with a dense top have also been used for inverse design [13, 77]. However, DNNs could have been utilized for the latter systems due to moderate number of degrees of freedom. The advantage of using CNNs here is in the detection of more complex patterns in the optical response data, which increases the efficiency of the obtained devices [77]. CNNs in photonics are also utilized for other purposes [70], some of which are discussed later in the text.

In Ref. [77], a CNN with a dense top was used to design a 1D photonic crystal slab, obtaining the geometrical parameters corresponding to a given band structure (Figure 3C). That inverse design has a one-to-many mapping nature, where, e.g., several photonic crystal geometries correspond to a single given band structure (raising some inconsistency issues). This is why some machine learning algorithms fail to converge when handling inverse design problems. The pre-trained forward model with fixed weights was connected to the inverse model to resolve this difficulty (Figure 3D) and the learnable parameters of the inverse model were trained to minimize the cost function defined as an error between the input band structure data *B*_*i*_ and the prediction of the forward model *B*_*j*_. Training the tandem neural network in such a way circumvents the nonuniqueness issue because the inverse model is not restricted to the production of prior specified designs from the training set [89]. Such an approach has been actively used in photonic inverse design to overcome inconsistency issues [19, 25, 89, 93, 94].

When the degrees of freedom in the design is thousands or greater, it is computationally more efficient to encode the input data to a reduced-dimensional space and reconstruct new designs from it [84]. Deep generative models are capable of doing so and creating new designs similar to the training set but with greater efficiencies. Deep generative models that have been actively used in the design of photonic devices include GANs (Figure 2C) [95–99], variation autoencoders [96, 100], [101], [102], [103], and global-topology-optimization networks [8, 104]. For example, in Ref. [73], an adversarial autoencoder was implemented to design a metasurface gap plasmon-based thermal emitter, which was a part of a thermophotovoltaic engine (see inset in Figure 3A), aiming to approach the limit of an ideal emitter (for which the emissivity is equal to one in the desired wavelength range and zero outside, left inset in Figure 3B), essential to reduce the unwanted heating of the photovoltaic cell occurring due to out-of-band radiation. The adversarial autoencoder consisted of three neural networks: an encoder, a decoder/generator, and a discriminator (Figure 3E). The encoder compressed the input 2D meta-atom pattern (64 × 64 binary image corresponding to a 4096-dimensional vector) into a reduced-dimensional space (15-dimensional latent space) using a DNNs with two hidden layers (512 neurons each). The discriminator was implemented to obtain the latent space within a pre-defined model distribution. The latter was made to represent the solution space continuously by a latent variable (specifically, a continuous Gaussian variable [105]). The decoder generated a 2D binary image of the meta-atom (4096-dimensional vector) out of its input 15-dimensional latent vector. The neural networks learned to obtain a continuous representation of the training data in the reduced-dimensional latent space. After training the adversarial autoencoder, new designs were generated by decoding the sampled latent vector. Then, the generated designs were refined using topology optimization and a CNN VGGnet (Figure 3A and B). The structure refinement smoothed the meta-atom patterns by ruling out sub-30-nm features and keeping the designs with the highest estimated efficiencies. The training set consisted of 8400 samples. Such a large dataset is typically required for adversarial autoencoder training. Generating a training set of such size using topology optimization is time-consuming. Thus, a set of 200 samples was obtained by topology optimization, and the actual training set of 8400 samples was generated by data augmentation. Specifically, the training set of 200 samples was expanded by 20 random lateral translations and a single 90° rotation of the meta-atoms. Thanks to the periodicity and symmetry of the metasurface thermal emitter structure, these perturbations did not affect the emissivity spectra. Such a method allowed for augmentation of the training dataset without additional full-wave simulations.

### 3.2 Image analysis in microscopy and nanoscopy

The analysis of experimental data is another area where deep learning has been successfully deployed. Such analysis is often time-consuming, requiring human input. With modern data acquisition techniques, the amount of experimental data can easily exceed what is feasible to analyze using conventional methods, making data analysis the limiting factor in many experiments. This is particularly true in microscopy. Every microscopy image may contain millions of pixels, and the designation of a simple rule connecting the individual pixel values to the desired output is non-trivial. Unsurprisingly, in the past decade, deep learning has created a new paradigm for the analysis of microscopy images, making precise, automatized, and objective data analysis possible at speeds orders of magnitude faster than for conventional methods. In addition, deep-learning-powered approaches have recently demonstrated the capability to extract information beyond the limits of traditional methods, making it possible to analyze data with unprecedented details.

**Figure 4:**
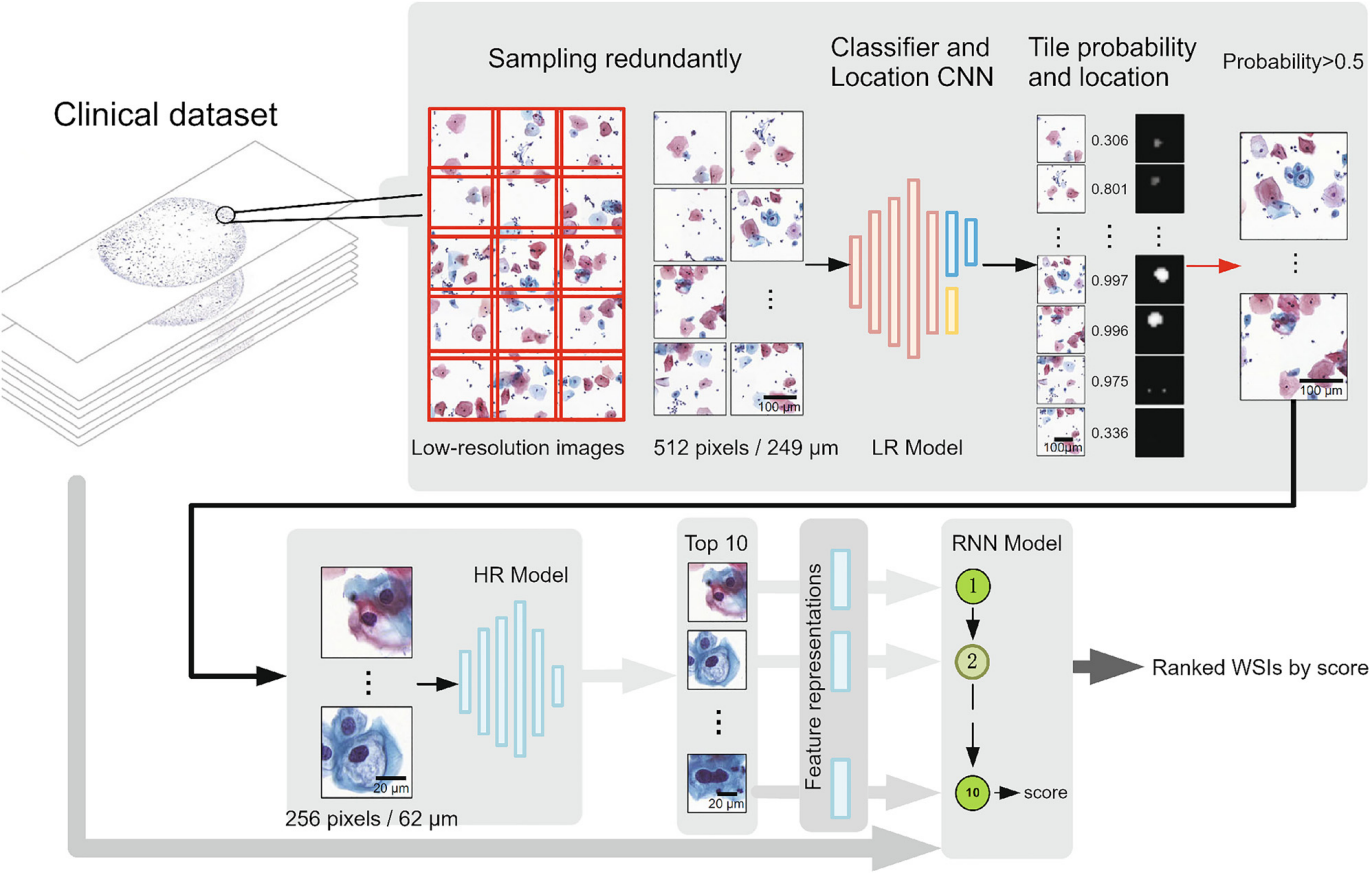
**Image analysis for a coarse-grained classification of histopathology slides using a CNN**. A low-resolution image of the entire slide is divided into non-overlapping tiles, which are independently passed through a low-resolution model that locates suspicious lesion regions. These regions are identified at high resolution by the high-resolution model that outputs a probability of the presence of a lesion cell in a tile. This high-resolution model outputs the ten most suspicious lesion tiles. Finally, these ten highest-scoring tiles are analyzed by an RNN to produce a final score for the entire slide. Image reproduced with permission from Ref. [107] (Copyright 2018 Springer-Nature).

The most well-known example of deep-learning-enhanced image analysis is that of image classification. The task is to classify objects in an image into predefined classes. The input is typically a cropped version of the whole input image, containing only a single object. The neural-network architecture is typically a CNN, which enables extracting object features at multiple scales with reasonable computational cost. As an example, Ref. [106] used a specific CNN architecture called Inceptionv3 to classify and predict mutations from lung cell histopathology slides. The neural network analyses nonoverlapping tiles in the image, providing a single classification score for each tile. The result is a downsampled image where every pixel corresponds to the classification of a specific tile. As another example, Ref. [107] proposed a deep-learning framework for whole-slide classification for cervical cancer screening. There, each slide can contain tens of thousands of cells. Therefore, the manual identification of lesion cells can be highly time-consuming. The authors propose a three-stage classification scheme: first, a CNN analyses a low-resolution image of the entire slide, indicating suspicious regions. After that, a second CNN analyses high-resolution images of the areas proposed by the first CNN and outputs a probability that the region contains a lesion cell. Finally, the ten highest-scoring regions are analysed by an RNN that provides a final score for the whole slide (Figure 4).

**Figure 5:**
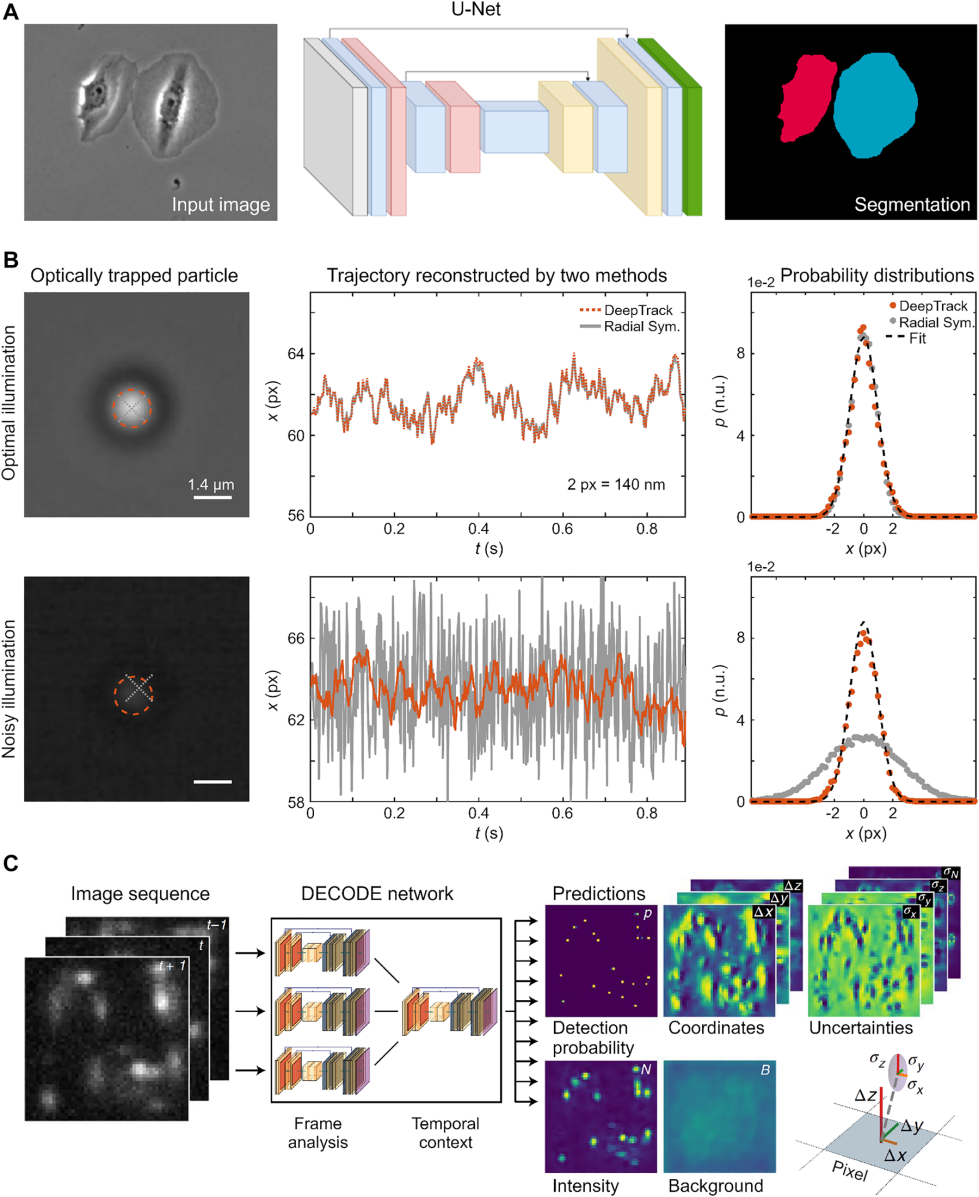
**Image analysis by neural networks**. **A** Pixel-wise classification to segment objects in microscopy images. The method is based on the U-Net architecture and outputs a binary image corresponding to cells (white) and background (black). **B** Particle localization using a CNN. The CNN is trained using simulated images of microscopic particles. The neural network outputs the position of a particle within a region of fixed size. This model (orange lines) outperforms traditional approaches (gray lines) in terms of accuracy, in particular for noisy images (lower panels). **C** Particle localization using a U-Net to identify the position and intensity of particles pixel by pixel. For each pixel, the network predicts the probability that an emitter exists near that pixel, the intensity of that pixel, the three-dimensional vector connecting the pixel to the closest emitter, as well as an estimate of the localization uncertainty. Panel A is reproduced with permission from Ref. [109] (Copyright 2019 Springer-Nature), panel B from Ref. [59] (Copyright 2019 Optica), and panel C from Ref. [111] (Copyright 2021 Springer-Nature).

Classification can also be performed on a pixel-by-pixel basis. One such example is image segmentation, where the task is to classify each pixel as either belonging to an object or to the background. This task is challenging using conventional image analysis techniques if the objects do not strongly contrast with the background, which is often the case for biological imaging. In such cases, the standard approach is the manual segmentation of the images. In 2015, Ref. [108] introduced the *U-Net architecture*, which enables efficient classification of each pixel in the image and automated segmentation of biomedical images [109] (Figure 5A). The U-Net architecture is a special type of CNN that takes an image as input and transforms it into another image. The segmentation of biomedical tissue images [109]) is achieved by first downsampling and then upsampling the image through a series of convolutional layers. Again, downsampling is performed to detect the features in the image and reduce the computational load, while upsampling is used to reconstruct the picture and carry out the segmentation. The downsampled images at the contracting part of the U-Net are concatenated with the images at the corresponding levels of the expanding part. This is performed to preserve local, high-resolution information about the picture. Another study employed a multichannel U-Net model to segment fluorescence images with heterogeneous marker combinations [110]. In particular, it showed that using an attention module makes the resulting model robust to the missing module problem, where only a limited subset of the marker combinations are available.

A further step in the analysis of microscopy images is to quantify the properties of the objects in the field of view. Examples include precisely identifying the location of objects in an image [59, 112], quantification of the scattering properties of objects from microscopic images [51, 113], and analysis of particle motion from sequences of images for characterizing anomalous diffusion [114] or the underlying force field [115]. A prime example of data regression is in object localization. Localizing objects in microscopic images has traditionally been based on thresholding techniques, in which adjacent pixels sharing similar intensities are grouped to form an assessment of where the objects are located in an image. The success of such pixel-by-pixel-based techniques requires the pixel-wise intensity of the objects to be well separated from that of the background. In many cases, such as in brightfield imaging or in the presence of noise, that assumption may not hold, making particle localization challenging. In contrast to the pixel-by-pixel-based thresholding analysis, CNNs instead extract features of images at multiple length scales. This enables the network to identify *spatial correlations* in the image data and to learn to exploit these correlations to classify the presence or absence of objects. This approach has been employed in various experimental situations and has enabled automated particle localization in challenging conditions. For example, in Ref. [59], the authors use a CNN to accurately determine the location of a single particle within a small image region (101 × 101 pixels). This approach was demonstrated to achieve higher localization accuracy than traditional algorithmic approaches, particularly under poor signal-to-noise ratios (SNR) (Figure 5B). This approach can be helpful for prediction refinement, assuming that some other method has already identified potentially interesting regions in the image. In Ref. [111], the authors instead used a U-Net to directly localize single-molecule emitters in an entire field of view, providing 3D localization as well as an estimate of emitter intensity in a single shot (Figure 5C).

### 3.3 Multimodal and transfer microscopy

**Figure 6:**
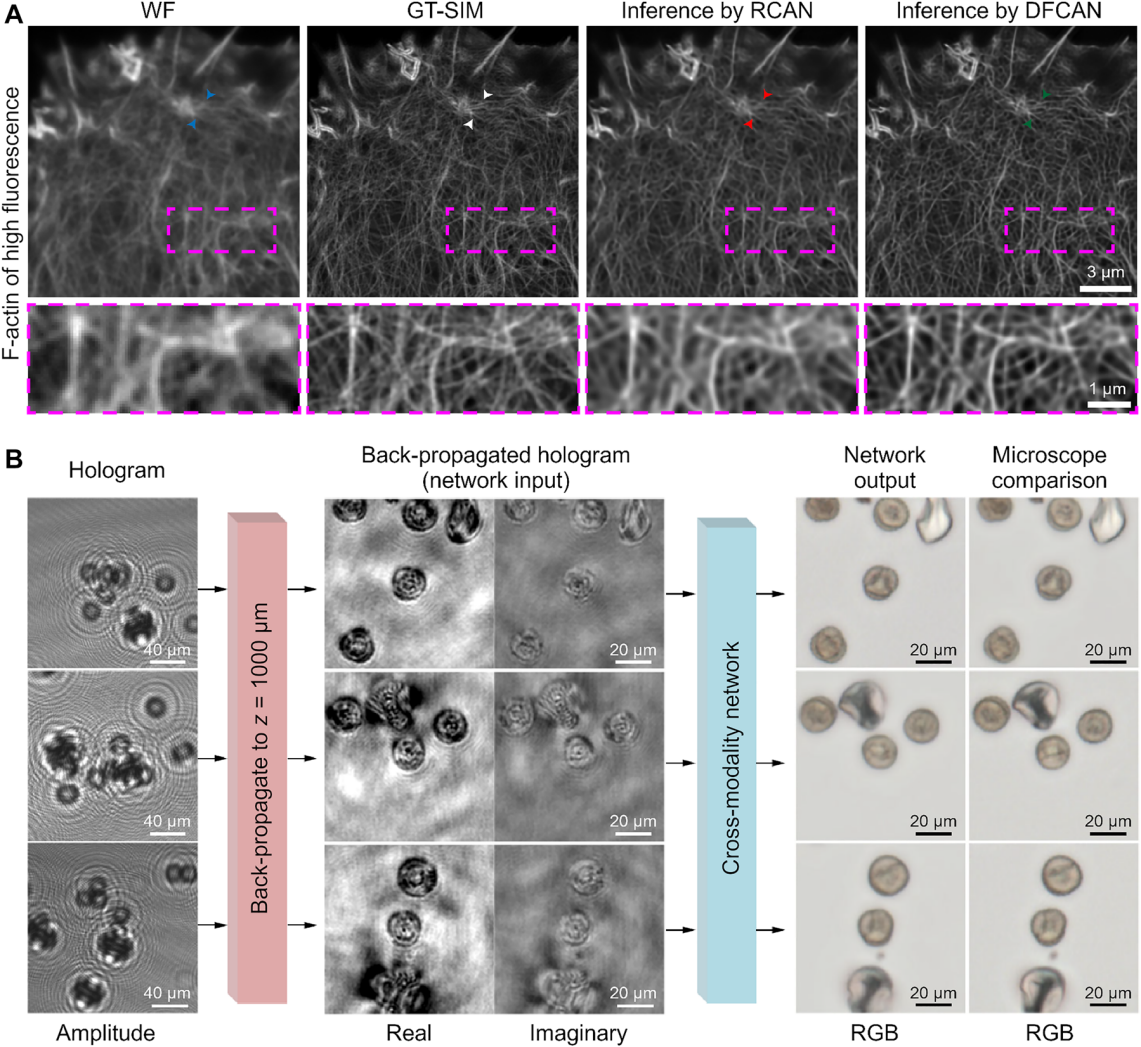
Multimodal and transfer microscopy. **A** Deep learning can be used to enhance the resolution of microscopy images. The core idea is to record sets of low-resolution images (the wide-field (WF) image exemplified in the leftmost panel) and high-resolution images (ground truth structured illumination microscopy (GT-SIM) image exemplified in the panel second to the left) of the same field of view, on the same optical setup. The neural network, often a GAN, is then trained to reconstruct the high-resolution image from the low-resolution image as input. The output examples are shown in the two rightmost panels, F-actins inferred by residual channel attention network (RCAN) and deep Fourier channel attention network (DFCAN). **B** A different way to utilize deep learning is by training models to convert between different microscopy modalities. In this example, a GAN was trained to predict brightfield images using inline holographic images as input. Panel A is reproduced with permission from Ref. [124] (Copyright 2021 Springer-Nature), and panel B from Ref. [120] (Copyright 2019 Springer-Nature).

Image classification and regression seek to reduce the information content of an image into a list of numbers. Beyond this, deep learning has proven effective in *image-to-image transformation*, in which the objective is to transform the input image into another image for further data processing. Examples include super-resolution imaging [116, 117], 3D volumetric imaging [118, 119], cross-modality transformation [120–122], and speckle pattern deconstruction [123]. As an example, in Ref. [116], a GAN (Figure 2C) was utilized to artificially enhance the resolution of optical microscopy images. The authors collected low- and high-resolution images of cells and nanoparticles. Notably, the low- and high-resolution images were obtained sequentially on the same field of view of each sample. The task of the neural network was to output the high-resolution image given the low-resolution one as an input. The network outperformed standard image deconvolution algorithms and matched the resolution of the optical method used to acquire the ground truth data. Furthermore, in Ref. [124], the authors demonstrated that by including network layers that analyze the Fourier spectrum of the input images, the details in the obtained super-resolution images were improved (Figure 6A). Deep learning can also be used to transform between different imaging modalities [120–122, 125, 126]. As an example, Ref. [120] used a GAN to transform holographic images into brightfield images, enabling volumetric imaging without the speckle noise typically associated with coherent imaging techniques (Figure 6B).

At this point, a word of caution is required. While deep-learning models can be trained to perform seemingly impossible transformations of the input data, these models cannot learn beyond the information content of the training data. In other words, deep-learning models can be trained to transform microscopy images from a high-information content image to a low-information content representation, while going in the opposite direction requires the model to extrapolate information based on knowledge it acquires from information in the training data. Utilizing such extrapolated data for further analysis and decision-making is risky, as it relies on information not present in the input data. Depending on the purpose of the analysis, it may be more robust to train a model to explicitly extract relevant features from the input image directly instead of first performing a potentially error-prone modality transformation.

## 4 Opportunities

Going beyond the success stories presented in the previous sections, deep learning remains underutilized in several areas. This section provides an overview of the areas where we believe that photonics and deep learning can still work synergistically to offer novel opportunities.

### 4.1 Quantitative data analysis

**Figure 7:**
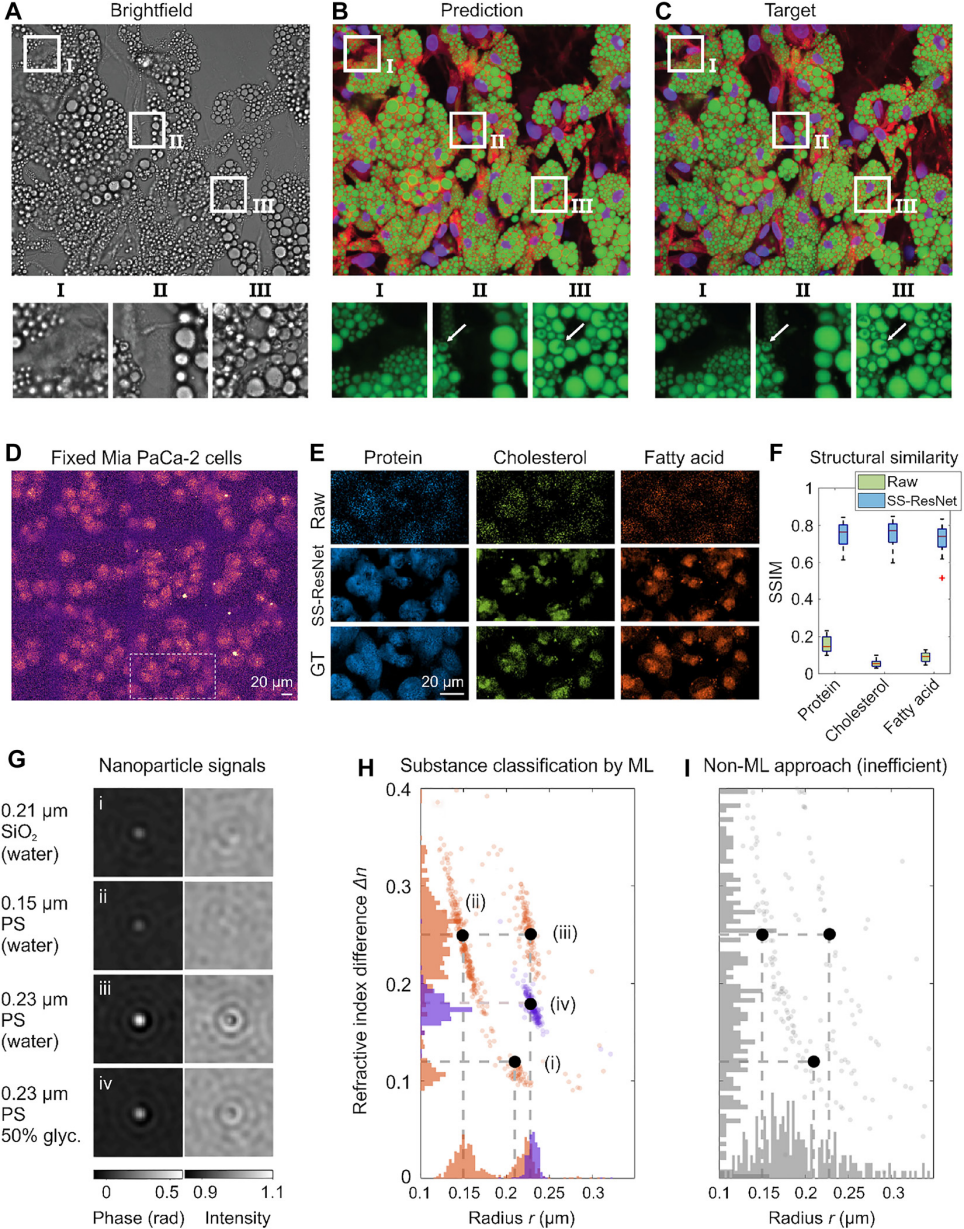
Quantitative imaging with deep learning. **A–C** Brightfield images of adipocytes **A** converted into virtually stained fluorescent images **B** and comparison with the relative chemically-stained ground truth **C**. The GAN was trained to reproduce images where lipid droplets (green), cytoplasm (red), and nuclei (violet) were separately stained. The resulting virtually stained structures were shown to quantitatively match the fluorescently stained structures in size and morphology, demonstrating that the network learns to distinguish different cellular structures in brightfield images based on their interaction with the illuminating light. **D–F** Raw Raman scattering signals from biological samples **D** retain quantitative information about the Raman spectrum of the sample, enabling the quantification of lipids and proteins in living cells using a U-Net **E**. **F** Structural similarity (SSIM) index for raw and spatial-spectral residual net (SS-ResNet) normalized by the ground truth of the three chemical channels. **G–I** Holographic images of nanoparticles contain quantitative information about the size and refractive index of the particles. This information can be decoded using deep neural networks to provide much-improved characterization accuracy compared to traditional methods. Panels A–D are adapted with permission from Ref. [122] (Copyright 2021 AIP), panels D–F from Ref. [127] (CC-BY), and panels G–I from Ref. [128] (Copyright 2021 ACS).

Deep-learning models can quantitatively measure the properties of their input data. This becomes particularly useful with several microscopy techniques that are today considered qualitative due to the difficulty of extracting quantitative information from light scattering data. For example, brightfield microscopy is arguably the most widely used microscopy technique found in essentially every scientific laboratory. However, due to the low image contrast and incoherent illumination, it is extremely challenging to extract quantitative information from brightfield images, so brightfield microscopy is commonly used as a qualitative technique. However, brightfield images are superpositions of scattering patterns formed by multiple wavelengths of light and contain vast amounts of information. For example, virtual staining of quantitative phase images [126] and brightfield images [122] has recently been demonstrated, transforming such images into synthetic fluorescence images where specific structures have been stained. Importantly, for brightfield imaging, the resulting structures have been shown to *quantitatively* reproduce both the morphologies and fluorescence intensities of the corresponding structures (Figure 7A–C) [122]. This procedure circumvents the limitations of brightfield imaging, namely its low image contrast and challenging data interpretation. Besides, it overcomes the limitations of fluorescence staining, namely potential toxicity, fluorescence bleaching effects, and variability in results between different professionals performing the stain, in this way transforming brightfield into a quantitative microscopy technique.

In other instances, the data itself may be quantitative, but the complexity of the data might make quantitative analysis challenging. For example, in hyperspectral imaging, each pixel contains a wide spectral profile that can be used to characterize an object. The vast amount of data available in each image makes it difficult to analyze it using conventional techniques. Ref. [129] uses a U-Net to predict the location of multiple drugs in a mouse liver from mass spectroscopic images, showing the potential of deep learning for the analysis of such complex data. Furthermore, Ref. [127] demonstrates stimulated Raman spectroscopic imaging of biological samples in the fingerprint region (400–1800 cm^−1^). Raman scattering in this spectral region provides “fingerprints” of the chemical composition of a sample and is, therefore, a highly useful characterization technique for many materials. For biological materials, the Raman scattering in this region is very weak, and it has been challenging to perform fingerprint Raman imaging on such samples. Using a U-Net architecture, the authors demonstrate an enhancement of the contrast of Raman spectroscopic images, which enables quantitative measurements of the lipid and protein contents of living cells (Figure 7D–F).

As a different example, the scattering patterns of nano- and microparticles contain information about their size, refractive index, and shapes. However, extracting these properties from experimental scattering patterns is challenging, as it requires solving the inverse problem for Maxwell’s equations. Instead, deep learning-powered solutions, which fit the Mie theory to the experimentally obtained scattering patterns, have enabled direct sizing and refractive index determination of particles with radii from about 100 nm to μm [113, 128] (Figure 7G–I).

As a final example, surface plasmon resonance is an optical effect occurring in metallic nanoparticles when they are irradiated. By measuring the absorbance of such nanoparticles as a function of the angle of incidence, the refractive index of the immediate surrounding of the nanoparticles can be determined. That is often used to characterize nanofilms and surfaces. However, when used as an imaging modality, the angle of incidence is kept fixed. The method is then used for qualitative investigations of surfaces rather than as a quantitative characterization tool. Ref. [130] demonstrates quantitative refractive index measurements for surface plasmon resonance imaging through the development of a deep learning-powered phase retrieval algorithm.

### 4.2 Nanophotonics for deep learning

Various systems are potential platforms for practical neuromorphic computing [53]. Some of the desirable aspects of using photonic-based systems instead of silicon floating-point units are their speed and low power consumption [131, 132]. Furthermore, photonic information processing systems are highly configurable and can process in parallel with amplitude, phase, polarization, and frequency [133], which naturally leads to a wide array of useable techniques. In this section, we will take a closer look at deep learning developments in photonics systems with a focus on systems that have been experimentally realized. They offer a good prospect for implementing neural networks, particularly with photonic chips and 3D printing [62, 134–141]. Our discussion will start with how non-linear activation functions are achieved in photonic systems, followed by the construction of fundamental neural network units such as perceptrons and multiply-and-accumulate (MAC) units, and finally, how these systems can be cascaded (layered) to implement deep learning.

A key element of sophisticated classical computing systems is stable and coherent switching behavior between two definite states. Neural networks are a model which incorporates non-linear switching behavior with linear interconnects. The choice of non-linear transfer functions changes from implementation to implementation and from application to application. One of the most common examples is the sigmoid function, which can be realized optically using a deeply saturated differentially biased semiconductor optical amplifier [142]. The pulse train in natural neural networks can be mimicked in systems that resemble the Hodgkin–Huxley circuit model [143]. In this model, the injected current is considered a phase-space parameter. Below the threshold current, the potential difference remains small, and no pulsing behavior is observed. At the threshold current, the dynamics of the circuit switches through a Hopf bifurcation, and periodic solutions can chaotically emerge. Passive optical amplifiers and cavities with parametric instability can also follow this behavior [144]. In general, any kind of bifurcation can be used [145]. These systems are considered for use in artificial neural networks because of support for Hebbian learning models (changes in neuron–neuron association with stimulation in time) in living organisms [146, 147].

The photonics-based schemes can be all-optical [137, 148–151] or electro-optical [140, 152–156] (see also reviews [157–159] for more details). The optical and electro-optical implementations of the activation functions in actual neural networks include phase change materials [137, 160, 161], Fano resonances in nanostructures [162], non-linear states (bifurcation) [145], wavelength-division multiplexing using optical amplifiers [142], electric-optic modulators [152–154], vertical-cavity surface-emitting lasers (VCSELs) [163, 164], passive mode locking with quantum dot [165], chip-based electro-optic feedback circuit [155], and Kerr non-linearity [139]. Only a few of these methods will survive in products in the future due to limits to the level of miniaturization that can be achieved for the corresponding physical process. Compared to the all-optical methods, electro-optical methods can be advantageous due to their relative simplicity in creating activation functions such as rectified linear units (ReLUs). Alas, this comes at the cost of additional waste heat production, limiting miniaturization. A comprehensive and more technical review of these different technologies can be found in Ref. [141].

Summation is an important part of data manipulation and transformation. The core functionality of a perceptron is to calculate an output that is a function of a weighted sum of its inputs. In CNNs [166], the data is multiplied element-wise with some kernel and summed. In RNNs [167], there is an implicit integration in the feedback. The details of how physical implementations of these data structures are made are important because they ultimately determine the speed, accuracy, and efficiency that make a computing system operate. In the single-mode domain, the summation of photons can be performed either by intensity or complex phase (vector/scalar field) [168]. For example, chip-based Mach–Zehnder interferometers have been used for vowel recognition [136] and could conceivably be adapted for general CNN and RNN tasks.

**Figure 8:**
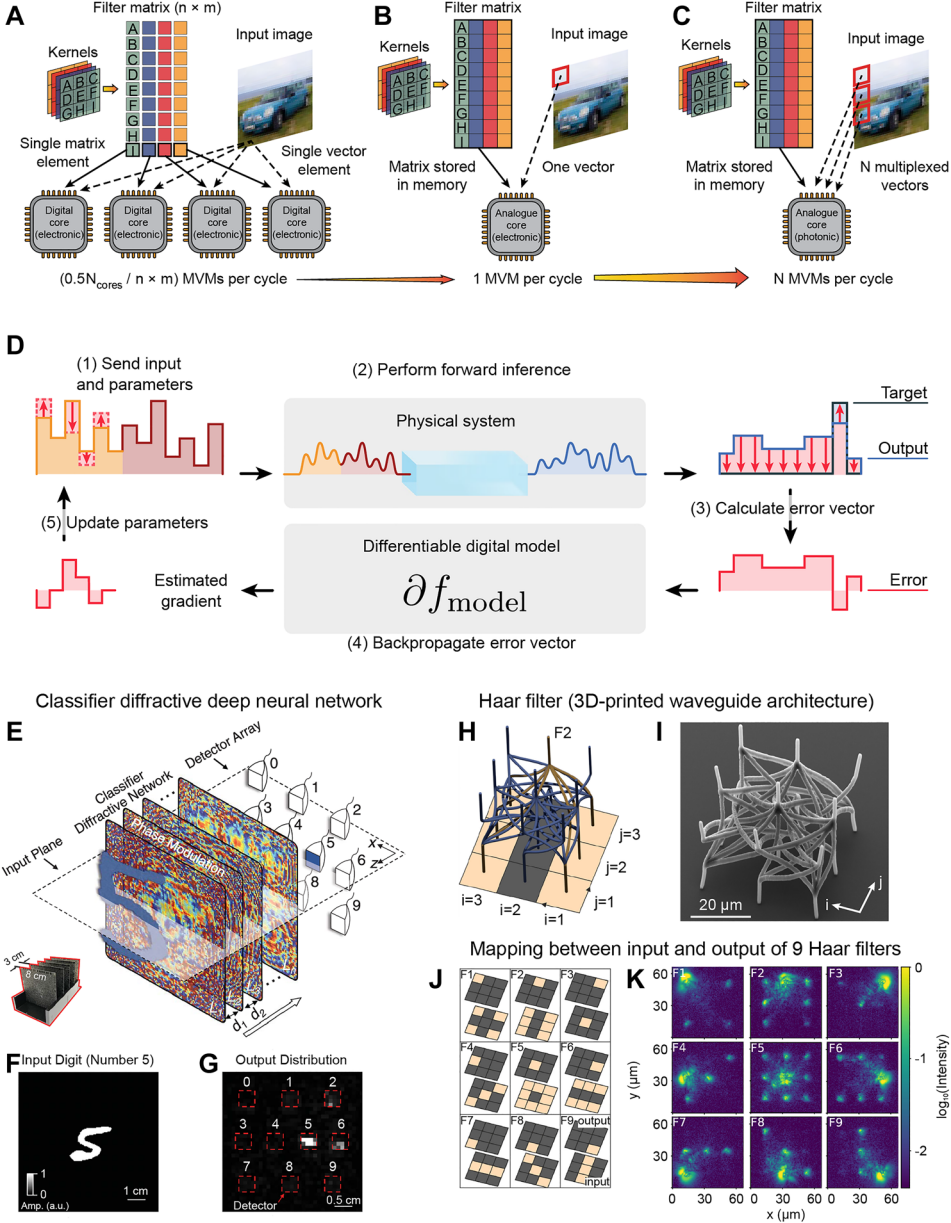
Deep learning with physical systems. **A** Digital electronics requires many sequential processing steps distributed across multiple cores to compute convolutional operations on an image. **B** In contrast, an entire matrix-vector multiplication (MVM) can be performed in a single step using analogue electronic in-memory computing. **C** Finally, in photonic in-memory computing, wavelength multiplexing is used to introduce an additional degree of freedom, enabling multiple MVM operations in a single time step (parallel convolutional processing using an integrated photonic tensor core). **D** Recently, it has been demonstrated that efficient training can be achieved by implementing error backpropagation using physics-aware training, where the forward pass in the training step is performed by the physical system, while error backpropagation is performed in a numerical system designed to mimic the response of the physical system. **E** Diffractive Deep Neural Networks comprise multiple transmissive layers, where each point on a given layer acts as a neuron with a complex-valued transmission coefficient. Here, a handwritten digit classifier that classifies **F** input digits (0, 1, …, 9) based on **G** 10 different detector regions at the output plane of the network, each corresponding to one digit. **H** Design and **I** SEM micrograph of a 3D-printed Haar filter with a kernel of width three. **J** Schematic illustration of the input–output mapping of nine Haar filters (F1–F9). **K** Optical characterization of the filter’s connection topology, injection at the output port, and recording the input ports emission. Panels A–C are adapted with permission from Ref. [169] (Copyright 2021 Springer-Nature), panel D from Ref. [170] (Copyright 2022 Springer-Nature), panels E–G from Ref. [134] (Copyright 2018 AAAS), and panels H–K from Ref. [135] (Copyright 2020 Optica).

Integrated photonics has the potential to perform vast parallel matrix multiplications. Currently, over a trillion (10^12^) MACs have been demonstrated with integrated photonics [140, 169]. By virtue of it being based on light (photons), which exhibits weak coupling in dielectrics, and the ease at which different frequency components can be separated, it has two potential advantages over electronic counterparts: such a system can use wavelength-division multiplexing to separate data into discrete streams of information, and it potentially exhibits much lower dispersion than electron-only systems, which enables high modulation rates. The wavelength-division multiplexing scheme and comparison with traditional computing for the same data is shown in Figure 8A–C.

Although neuromorphic computing has been shown to greatly accelerate and reduce the energy requirements of the inference stage of deep learning models, the actual training of such computing systems has proven to be a challenge. One reason for this is that the backpropagation algorithm, which is the pervasive algorithm for training neural networks, cannot be implemented directly in a physical system. This challenge has been recently overcome by combining physical systems with a numerical model which emulates the behavior of the physical system, demonstrating efficient training of physical neural networks using backpropagation [170] (Figure 8D).

In a free-space optical system, convolutions come naturally out of considering the equations of light propagation where the propagation kernel itself is a convolution. This provides an opportunity to produce a deep neural network based on the printing of successive complex amplitude filters, such as demonstrated for a diffractive deep neural network (D2NN) [134] (Figure 8E–G). In this work, a machine learning model of a physical system was first modeled on a computer, and the resulting stacked complex filtering structures were printed using lithographic techniques. This neural network was able to identify objects within images encoded on light projected through the complex photonic structure [134]. Furthermore, convolutional image processing, including classification using deep neural networks, has been demonstrated, e.g., in Refs. [134, 169, 171].

Micro 3D printing can create Cantor-set-like photonic circuits with a 3D structure that allows deeply connected networks to occupy less space than a traditional printed photonic circuit [135] (Figure 8H–K). These structures were shown to exhibit a high degree of convolution in a small space, which provides a potential avenue for extremely compact CNNs.

### 4.3 Microscopic particles with embodied intelligence

Natural systems have evolved powerful sensing capabilities to gain information about their environments and to communicate [172, 173]. For example, in swarms of midges, schools of fish, and flocks of birds, individuals exchange information as part of their behavior to self-organize into a collective state [174]. Microorganisms have also developed complex strategies to survive and thrive in their environment by integrating sensors, actuators, and information processing. Their biochemical networks and sensory systems are optimized to excel at specific tasks, such as climbing chemical gradients [175], coping with ocean turbulence [176], and efficiently foraging for food [177, 178]. They have also acquired complex strategies to interact with their environment and with other microorganisms, leading to the emergence of macroscopic collective patterns. These patterns are driven by energy conversion from the smallest to the largest scales and permit microorganisms to break free of some of their physical limits. For example, dense systems of bacteria develop “active turbulence” at length scales where only laminar flows are expected from the underlying physical laws [179, 180]. As another example, dense filaments and motor proteins, which are the structural building blocks of cells, develop active nematic structures with new physical properties [181, 182].

On the other hand, synthetic microscopic systems that try to emulate living systems still present many fewer possibilities. Most experimental studies have been constrained to steric, electrostatic, phoretic, or hydrodynamic interactions, which are readily available from physical interactions [5]. Even these simple interactions can lead to interesting complex behaviors and self-organization whose onset is often observed in artificial systems where increased energy input above a threshold density drives a phase transition to an aggregated state. An example of such behaviors is the formation of “living crystals,” which are metastable clusters of active particles [183, 184].

**Figure 9:**
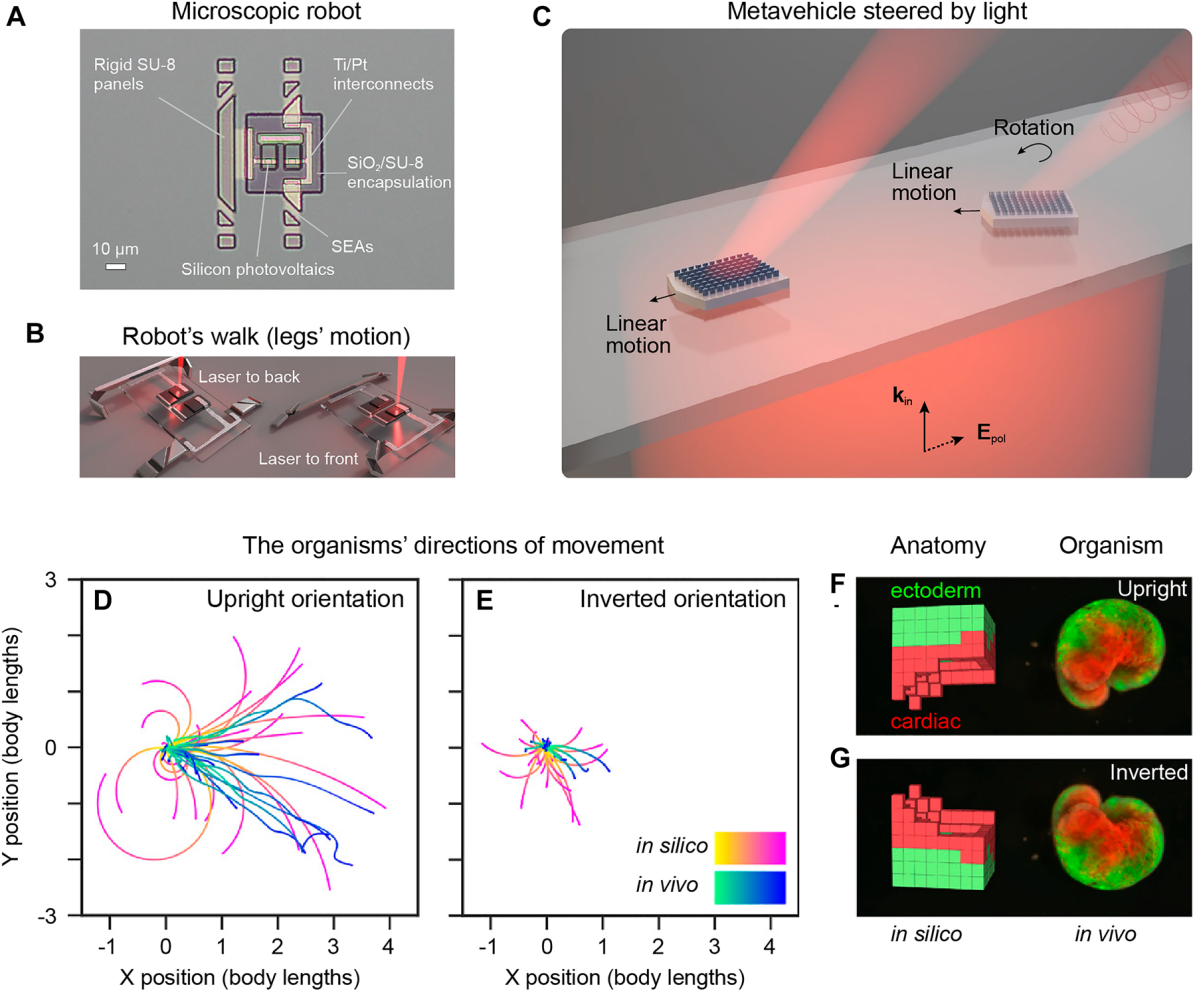
Toward embodied intelligence in microscopic particles. **A** Optical image of a microscopic robot. It has two parts: a body with internal electronics and legs that actuate. The electronics are simple circuits made from silicon p–n junctions and metal interconnects, encapsulated between a layer of silicon dioxide and a layer of SU-8 photoresist. The legs are made from a new class of voltage-controlled surface electrochemical actuators (SEAs) and rigid photoresist panels. The panels control the folded shape of the leg while the SEAs produce motion. **B** By directing laser light to photovoltaics that alternately biases the front and back legs, the robot walks along patterned surfaces. **C** By incorporating an engineered metasurface into a microparticle, the changes in linear and angular momentum of the incident light can be employed to propel and steer the microparticle across a surface, realizing a metavehicle. **D–G** Advanced in silico design using artificial intelligence is employed to generate multicellular microorganisms with specific behaviors. Panels A–B are reproduced with permission from Ref. [185] (Copyright 2020 Springer-Nature), panel C from Ref. [186] (Copyright 2021 Springer-Nature), and panels D–G from Ref. [187] (Copyright 2020 NAS).

Photonics has the opportunity of making microscopic particles intelligent, providing the tools for artificial microscopic particles to acquire, elaborate, and respond to information from their environment [5]. This can be made by different means. For example, Ref. [185] has recently developed a lithographic fabrication-and-release protocol to build microscopic walking robots activated by light, which provide a new class of voltage-controllable electrochemical actuators that operate at low voltages (200 μV), low power (10 nW) and are completely compatible with silicon processing (Figure 9A). This permits the authors to realize microscopic particles that can be actuated by shining a beam of light on them, as shown in Figure 9B. Also, Figure 9C shows another recent example where microscopic particles are enhanced with metasurfaces that alter the linear and angular momentum of the incident light, therefore, permitting steer the particles [186]. However, these approaches only provide photonic-actuated microrobots, which still require some external control and feedback.

In this context, machine learning can provide new approaches to realize these possibilities. In fact, the early studies presented above have mainly relied on designs based on human intuition, which is now leading to diminishing returns. Designs obtained by machine learning can go beyond what can be simply imagined by human intuition and therefore open new possibilities. For example, machine learning can help to achieve onboard sensing and decision-making, as opposed to external computer-controlled feedback loops. This has been recently demonstrated in a proof-of-principle study by designing reconfigurable organisms [187], as shown in Figure 9D–G. In this work, artificial intelligence methods were employed to automatically design diverse candidate lifeforms in silico to perform some desired function, and transferable designs were then created using a cell-based construction toolkit to realize living systems with the predicted behaviors. In the future, these reconfigurable living organisms can be further enhanced with photonic capabilities such as lasing [188] and light guidance [189].

## 5 Challenges

In the previous sections, we have shown the potential of deep learning to enhance the study of light–matter interactions beyond the capabilities of conventional methods, providing a fast, automatized, and noise-resilient route to optimize the output of optics and photonics experiments. However, in designing, executing, and validating the performance of deep-learning-based methods, one is faced with several considerations and challenges distinct from those experienced when employing conventional methods. This section will review the essential challenges and provide simple guidelines for the effective deployment of deep-learning-based techniques in the study of light–matter interactions.

### 5.1 Training data augmentation and simulation

The first challenge is to obtain high-quality training data. This is a challenge common to all supervised machine-learning methods and involves the generation of matching input/output pairs. For the network to generalize to unseen data, the network must learn to recognize relevant features of the input data. This requires that the input data represent the full range of expected cases where the trained network will be applied. Determining whether the input data is sufficiently general can be non-trivial, particularly in cases where the physical rules connecting input and output are unknown. In addition to acquiring a representative set of network inputs for the training, the corresponding target outputs need to be constructed. For example, for image classification and segmentation, the target outputs are often built through manual labeling, which is a time-consuming process, limiting the amount of training data that can be constructed within a reasonable amount of time and effort. Further, the use of user-labeled data for network training makes the network output subjective, as it reflects the biases of the user constructing the labels.

Data normalization and augmentation are often employed to reduce the amount of data required for network training. Data normalization aims at making the data easily interpretable by a neural network. While data normalization does speed up the learning process of neural networks, it also implies a loss of data, which might be of relevance, particularly for regression tasks. Therefore, choosing the right type of data normalization, which quantitatively retains the information of interest, is crucial for successful deep learning deployment in science. A trivial but sometimes overlooked example is that the sample-to-sample variability within a dataset is lost when individual samples from that dataset are independently normalized. In cases where such variability is expected to be important, e.g., for quantitative applications, a global normalization across the entire dataset is to be preferred.

The purpose of data augmentation is to perform multiple transformations to the input, which have a predictable effect on the expected network output. For example, it is possible to generate multiple training images from a single input image, thereby extending the available training set by image rotations, scaling, and cropping. However, one should be wary of transformations that do not necessarily conserve the relation between the input and output, particularly in cases where the underlying theory connecting input and output is unknown. For example, in most cases, the analysis of microscopy images is invariant under translations and rotations. However, depending on the context, transformations that alter the pixels themselves, such as scaling or elastic transformations, may introduce unpredictable artifacts in the analysis. As a general rule, one should only employ augmentations that have a predictable effect on the analysis.

One way to partially overcome these challenges is to use simulated data for training neural networks. This approach can generate data on the fly during training, enabling essentially unlimited training data. Nonetheless, verifying that the training data represents the experiment realistically is still non-trivial. Essentially, the simulation must be sufficiently exact to capture the relevant features in the experimental data accurately. As an illustrative example, in Ref. [59], simulated microscopy images of single particles were used to train a network to estimate the position of particles within an image. Since the experimental data consisted of particles whose intensity profiles could be well described by Bessel functions, training the network on combinations of two-dimensional Bessel functions with varying width, position, and intensity was sufficient to provide good localization accuracy. In contrast, in Ref. [128], simulated data were used to train a network to quantify the size and refractive index of individual nanoparticles from their scattering patterns. In this case, the relevant information is encoded in the Fourier spectrum of the scattered field, and in order to capture the relevant features of the scattering patterns, the simulated data needed to consist of simulated Mie scattering patterns of particles passed through a synthetic replica of the experimental optical system including its aberrations.

Sufficiently complex deep-learning models can learn sophisticated correlations in the input data. The models can also be sensitive to out-of-distribution shifts. Slight changes in the parameters of the microscope in the last example would invalidate the trained model and require retraining. Detecting and making deep learning solutions robust to such perturbations is an active field of research, and multiple strategies have been proposed to partially address this issue [190, 191].

### 5.2 Architecture and hyperparameter optimization

The choice of network architecture is currently more of a form of art than an exact science. In general, if there exist a transformation connecting the input data to the desired output, any network architecture with sufficiently many adjustable parameters will be able to learn an approximation to this transformation. What can vary between different choices of architectures is the accuracy of the approximation, the training time, the training data amount required to reach this approximation, and the execution speed of the deployed network. What matters the most for the choice of network architecture and hyperparameters is the amount and type of available data and the desired output type. More specifically, inherent symmetries in the input data can often be used to guide the choice of network architecture. For example, when detecting and/or classifying objects in an image, the spatial location of the object within the image rarely matters for classification. Convolution kernels are intrinsically translationally invariant and are thus routinely used for such tasks. Beyond this, physically informed neural networks provide a strategy for imposing physical symmetries and constraints on the network prediction, typically by penalizing predictions that do not obey the specified symmetries [192].

In this way, the architecture of a neural network carries an inductive bias, which determines what relations and features of the data are most easily learnt and prioritized by the network [193]. Being aware of these biases can aid the development of efficient network architectures for solving a specific problem.

Regarding the choice of hyperparameters, such as the depth of the network and the number of adjustable parameters, this will typically depend on the amount of available data and the type of transformation the network is to learn. The deeper the network, the more complex changes the network can learn. On the other hand, if the available data is limited, there is a chance of overfitting the training data. Choosing the right balance requires iterative training with varying hyperparameters while carefully validating the network results on a large test set. It is often a good practice to start by training a relatively small network and keep increasing its size as long as its performance increases without overfitting the training data.

An open challenge is automatically determining mechanisms to define the best architecture and hyperparameters. For example, that has been done using some evolutionary architecture such as the neuroevolution of augmenting topologies (NEAT) genetic algorithm developed in 2002 [194] and its subsequent derivations [195]. However, such approaches have turned out to be quite slow in convergence and require many computational resources.

### 5.3 Performance benchmarking, validation, and reproducibility

Any use of deep learning in science needs to be motivated by a superior performance compared to standard methods by some predefined metric. Depending on the application, such metrics may include analysis speed, accuracy, or robustness to noise.

In some cases, such benchmarking is straightforward. For instance, for the inverse problem solvers, the viability of the obtained solution can be checked by explicitly solving the forward problem with the obtained solution as an input. However, in many cases, deep learning solutions are applied to situations beyond the capabilities of existing techniques and where no theory exists that maps the network prediction to the targeted output. Validating the output of the network in such cases poses a considerable challenge. To our knowledge, the best practice is to generate an experimental data set that can be analyzed using some traditional method. Once the deep learning solution has been validated against the traditional method on this data set, the next step is to generate a synthetic data set that simulates the experimental setup and where the ground truth is known perfectly. The quality of the synthetic data set can be evaluated by comparing the output of the traditional method and deep learning solution to the simulated ground truth for a range of parameters where the conventional method is known to perform well. Once the simulation quality has been validated, the final step is to tune the parameters in the synthetic data set to the instances where the traditional method fails and again validate the deep learning solution against the simulated ground truth for the updated data set. In this way, the deep learning solution can be shown to perform comparably to a traditional method for the cases where the traditional method is expected to work. Furthermore, the deep learning solution should outperform the traditional method on the synthetic data sets as well.

Such careful benchmarking and validation become particularly important when adversarial approaches are used to manipulate the data [196]. As we have seen in the previous sections, adversarial networks are trained to generate synthetic data based on some input and are designed to *look* realistic rather than to represent the ground truth fairly. Therefore, adversarial approaches are at risk of extrapolating the input data, effectively making up non-existing data [196]. There is an increasing awareness of these issues in the machine learning community, and several recent studies demonstrate that advances in image reconstruction and image analysis, provided by the use of GANs, can also be achieved without adversarial learning [118, 124, 197].

Finally, a related issue is the result reproducibility by the deep learning-powered analysis. In particular, the published deep learning solutions are typically made for specific data sets; therefore, they are unlikely to provide valid results when directly employed by a different research group on a similar, but not statistically identical, data set. Thus, there has lately been a surge of interest toward the development of publicly accessible software for customizing deep learning solutions without the steep learning curve commonly associated with machine learning [51, 198]. A set of guidelines were recently proposed for enhancing the reproducibility of the deep learning techniques [199]. As a minimal requirement, the data, model, and analysis code should be made publicly available (bronze standard). To meet the silver standard, authors should make the dependencies of the analysis installable with a single command, with the code properly documented. Further, all random components of the analysis should be made deterministic for reproducibility. Finally, the authors should make the full analysis reproducible with a single command to meet the gold standard.

## 6 Conclusions and guidelines

In this review, we have presented the current state and future perspectives for the application of deep learning in the fields of optics and photonics. Owing to the challenges related to validating and reproducing deep learning results, a set of community-wide recommendations for deep learning reporting and validation in biology was recently published [200], with the acronym DOME (**D**ata, **O**ptimization, **M**odel, **E**valuation). The recommendations are summarized as a checklist of questions that should be addressed when reporting deep learning results. These recommendations largely apply to the fields of optics and photonics as well; namely, every deep learning application should be able to provide answers to these questions:

– **Data:** How large is the data set used for training the model? How large is the validation set? Are validation and training set independent? Is the distribution of data different in the training and validation sets? Has the data set been used previously? Are the data publicly available?
– **Optimization:** What type of deep learning algorithm was used? Is the algorithm new? If so, why was it chosen over existing algorithms? Does the model use output from other deep learning algorithms as input? How were the data encoded and preprocessed prior to prediction? How many parameters does the model consist of? Was the feature selection performed? If so, how? Are the number of parameters much larger than the number of data points in the training set? If so, how was the overfitting ruled out? If not, how was underfitting ruled out? Are the hyperparameter configurations, optimization schedule, model files, and optimization parameters reported?
– **Model:** Is the model black box or interpretable? If the model is interpretable, can you give clear examples of this? Is the model classification or regression? What is the typical execution time? Is the software publicly available?
– **Evaluation:** How was the method evaluated? Which metrics were used for the evaluation? Was a comparison to standard algorithms made on benchmark data sets? Was a comparison to simpler baselines performed? Are the raw evaluation files available?

Finally, we remark that, beyond a large range of applications that have already been successful, there are still many fields where deep learning can have a large impact and, therefore, need to be explored. First, the execution speed of trained deep learning models provides new possibilities for experiments through the automatization of current setups. This can free these experiments from the need for continuous human intervention and supervision, permitting the acquisition of large-scale statistics that currently would be prohibitively work-intensive. In turn, this will allow scientists to study also relatively rare events that human operators on small sample sizes might disregard as outliers (e.g., in biomolecule pulling experiments using optical tweezers). Second, there is a great drive towards understanding not only *what* deep learning can do but also *how* it achieves it. This means gaining insights into the understanding and interpreting the black box that deep learning often represents. Once this work advances, it will open new possibilities to discover the theory underlying various phenomena by observing what the network learns. Third, nanophotonics can provide essential tools to make physical implementations of neural networks, which have remarkable advantages in terms of increasing computational speed and minimizing power consumption. In fact, as we have seen in the previous sections, there are already several proposals along these lines. One of the critical issues in this field is integrating these new neuromorphic computing technologies with current computational technologies based on Boolean electronic circuits. Fourth, arguably the holy grail of the field would be to employ machine learning concepts and techniques to realize microscopic particles capable of real intelligent behavior by autonomously processing and responding to information from their environment.
